# 492. Dectin-1 Stimulation Promotes a Distinct Inflammatory Signature in the Setting of Aging and HIV-infection

**DOI:** 10.1093/ofid/ofac492.550

**Published:** 2022-12-15

**Authors:** Heidi Zapata, Archit Kumar, Jiawei Wang, Albert Shaw, Haowen Zhou, Christopher Radcliffe, Lydia A Barakat, Brent Vander Wyk, Heather Allore, Hongyu Zhao, Sui Tsang, Data Manager, Subhasis Mohanty

**Affiliations:** Yale School of Medicine, New Haven, Connecticut; Yale School of Medicine, New Haven, Connecticut; Yale School of Medicine, New Haven, Connecticut; Yale School of Medicine, New Haven, Connecticut; Yale University, New Haven, Connecticut; Yale School of Medicine, New Haven, Connecticut; Yale School of Medicine, New Haven, Connecticut; Yale School of Medicine/Program on Aging, New Haven, Connecticut; Yale School of Medicine/Program on Aging, New Haven, Connecticut; Yale School of Public Health, New Haven, Connecticut; Yale School of Medicine/Program on Aging, New Haven, Connecticut; Yale School of Medicine/Medicine, New Haven, Connecticut

## Abstract

**Background:**

Dectin-1 is an innate immune pattern recognition receptor that recognizes and binds b-1,3/1,6 glucans on fungal pathogens.

**Methods:**

We evaluated Dectin-1 function in innate immune cells (Monocytes and Dendritic cells) in a cohort of HIV-positive and HIV-negative young and older adults (n= 81). PBMCs were stimulated with whole glucan particles (1,3/1,6-b-glucan, WGP), a specific Dectin-1 agonist and analyzed via intracellular cytokine staining (ICS) and multicolor flow cytometry of monocytes and dendritic cells. Sorted CD14+ CD16+ monocytes stimulated with WGP and subjected to RNA-seq analysis.

**Results:**

Stimulation of monocytes with β-D-glucans induced a pro-inflammatory phenotype in monocytes of HIV-infected individuals that was characterized by increased levels of IL-12, TNF-a, and IL-6, with some age-associated cytokine increases also noted. Dendritic cells showed a striking HIV-associated increase in IFN-a production. This increase in cytokine production paralleled increases in Dectin-1 surface expression in both monocytes and dendritic cells that were noted with both HIV and aging. Differential gene expression analysis showed that HIV-positive older adults had a distinct gene signature compared to other cohorts characterized by a robust TNF-a and coagulation response that was already increased at baseline, and a persistent IFN-α and IFN-γ response. HIV-older adults also demonstrated an activated dendritic cell signature and M1 macrophage signature upon stimulation that was not seen in young individuals. Pathways upregulated in all cohorts were the signaling pathways MTORC-1, hypoxia pathway (pathways involved in trained immunity) and the unfolded protein response. Of note MTORC-1 signaling was upregulated at baseline in HIV-Older adults.

**Conclusion:**

Overall, our study demonstrates that increased age and HIV-infection can affect the function of Dectin-1 and lead to a distinct inflammatory signature. Dectin-1 stimulation in HIV-Older adults led to a more pro-inflammatory phenotype that was demonstrated through both protein and gene expression. Dectin-1 induced inflammation in HIV-infected individuals may be contributing to the pro-inflammatory environment that is seen in the setting of both aging and HIV-infection.

**Disclosures:**

**All Authors**: No reported disclosures.

